# What maternal morbidities are and what they mean for women: A thematic analysis of twenty years of qualitative research in low and lower-middle income countries

**DOI:** 10.1371/journal.pone.0214199

**Published:** 2019-04-11

**Authors:** Isabelle L. Lange, Atf Gherissi, Doris Chou, Lale Say, Veronique Filippi

**Affiliations:** 1 Maternal Adolescent Reproductive and Child Health Centre (MARCH), London School of Hygiene and Tropical Medicine, London, United Kingdom; 2 University of Tunis El Manar, Tunis, Tunisia; 3 Department of Reproductive Health and Research, World Health Organization, Geneva, Switzerland; 4 Department of Infectious Disease Epidemiology, Faculty of Epidemiology and Population Health, London School of Hygiene and Tropical Medicine, London, United Kingdom; USC Keck School of Medicine, Institute for Global Health, UNITED STATES

## Abstract

**Background:**

With an estimated 27 million annual incidents of maternal morbidity globally, how they are manifested or experienced is diverse and shaped by societal, cultural and personal influences. Using qualitative research to examine a woman's perception of her pregnancy, its complications, and potential long-term impact on her life can inform public health approaches and complement and inform biomedical classifications of maternal morbidities, historically considered a neglected dimension of safe motherhood. As part of the WHO’s Maternal Morbidity Working Group’s efforts to define and measure maternal morbidity, we carried out a thematic analysis of the qualitative literature published between 1998 and 2017 on how women experience maternal morbidity in low and lower-middle income countries.

**Results and conclusions:**

Analysis of the 71 papers included in this study shows that women’s status, their marital relationships, cultural attitudes towards fertility and social responses to infertility and pregnancy trauma are fundamental to determining how they will experience morbidity in the pregnancy and postpartum periods. We explore the physical, economic, psychological and social repercussions pregnancy can produce for women, and how resource disadvantage (systemic, financial and contextual) can exacerbate these problems. In addition to an analysis of ten themes that emerged across the different contexts, this paper presents which morbidities have received attention in different regions and the trends in researching morbidities over time. We observed an increase in qualitative research on this topic, generally undertaken through interviews and focus groups. Our analysis calls for the pursuit of high quality qualitative research that includes repeat interviews, participant observation and triangulation of sources to inform and fuel critical advocacy and programmatic work on maternal morbidities that addresses their prevention and management, as well as the underlying systemic problems for women’s status in society.

## Introduction

While maternal mortality is a global development indicator that defines a country’s status in terms of maternal health [1], maternal morbidity has traditionally been left out of public debates. The reasons for this are complex and include a lack of its prioritization in national and international communities, compounded by the difficulties surrounding its definition and measurement [2]. Overall estimates of maternal morbidity are unknown, but even if we consider only morbidity episodes from the five main direct obstetric causes of maternal mortality, 27 million annual episodes were estimated to have occurred in 2015 based on a systematic review, reflecting “inequities in wealth, rights, and access to care” ([3] p.8). These inequities can negatively impact women, their families and communities, with economic, social, physical and mental repercussions [4–6]. Women’s experiences of maternal morbidities are central to assessing their significance and the role these conditions and broader determinants play in pregnancy and postpartum periods, and beyond.

The World Health Organization’s Maternal Morbidity Working Group (WHO MMWG), convened to draw attention to and develop methods for the measurement of maternal morbidity, recently defined maternal morbidity as “any health condition attributed to and/or complicating pregnancy and childbirth that has a negative impact on the woman’s well-being and/or functioning” [7]. ‘*Well-being*‘ and ‘*functioning’* take the definition beyond narrow clinical concerns, communicating that maternal morbidities are shaped by individuals’ social and physical environments and thus probably are more multifaceted and differently experienced than suggested by clinical diagnoses.

With the shift from the Millennium Development Goals (MDGs) to the Sustainable Development Goals (SDGs) in 2015, the targets for reproductive health and gender equality (SDGs 3 and 5) encourage action on improving maternal health outcomes, including maternal morbidity. Especially in lower income countries, researching and monitoring the burden of, and experience around, maternal morbidity can help governments develop strategies to improve health care services and to address the structural contributions to morbidities. This becomes increasingly important, as while maternal mortality decreases in most parts of the world, evidence fails to show similar declines for maternal morbidity [3].

Most efforts to consolidate findings on maternal morbidity in lower income countries have focused on measureable, quantitative evidence (for example [8–10]), while some qualitative reviews have focused on more narrowly defined morbidities in higher income countries (for example [11,12]). This has left gaps in understanding the impact on women’s lives in lower income countries which can face different, albeit diverse, contextual characteristics. Reflections on women’s perspectives need to take a qualitative approach, one that is scientific and centers on a woman’s experience of her conditions and the factors that influence how she is affected. Several authors recommend taking a lifecycle approach [13,14]. This is useful as a means of better understanding maternal morbidities, as it involves considering women beyond their pregnancies and considering life circumstances, including cultural and social factors and consequences when documenting changes she might go through over time. Qualitative research can take into account societal and cultural influences that may impact the way a morbidity is manifested or experienced.

We carried out a thematic analysis and synthesis of qualitative literature on women’s experiences of maternal morbidity between and including 1998 and 2017 in low and lower-middle income countries to understand the contribution of research on this topic and its potential for shedding light on maternal well-being in relation to women’s lives. Below, we analyze the research profile in terms of types of conditions, maternal stages researched, methods used, and geographical distribution. Through the process of identifying, ordering and analyzing the papers, we describe the most frequently addressed topics and examine themes that arise, exploring how they are addressed across different contexts and populations, and illustrate the influence maternal morbidities have on women’s economic, physical, social and inner lives. We close with reflections on the implications of these findings for the programmatic and research agendas surrounding maternal morbidities.

## Methods

### Overall approach

Scholars debate whether the principles of quantitative systematic reviews apply to reviews of qualitative literature [15]. Quantitative systematic reviews aim to answer a specific question, reduce the bias in the selection and inclusion of studies, appraise the quality of relevant studies, and summarize them objectively [16]. Some argue that a similar synthesis of qualitative data ruins the integrity of the original studies and separates out unifying themes at the cost of the studies’ ‘thick’ description [17]. The results of qualitative research are specific to a particular context, time and group of people, so attempts to generalize or combine findings can lead to the loss of contextualisation [18] and the danger of drawing erroneous conclusions. However, synthesis is basically a comparative method, which formed the backbone of anthropological and sociological research from the 1800s and has a long tradition of generating questions and insights across contexts and time. Thus the logical approach for conducting qualitative reviews is to employ qualitative methods [19], where the benefits of qualitative meta-syntheses include “integrations that are more than the sum of parts in that they offer novel interpretations of findings […]” [20].

We conducted a systematic review of the literature and employed a thematic analysis to explore and synthesize included articles. We chose this more open analysis approach to account for the diversity of the included articles which encompassed a heterogeneous range of styles, methods, and subtopics. As the methods for conducting a thematic analysis for systematic reviews can be inexplicit [18], we sought inspiration from other documented methods, most notably narrative synthesis, resulting in a hybrid approach. Narrative synthesis is rooted in a story-telling approach that seeks to generate new insights or knowledge by systematically and transparently integrating existing research findings [21]. It entails identifying key themes arising from multiple studies, summarising their findings, and looking across the body of work to develop a narrative that encompasses these themes. In our analysis, we noted authors’ key findings and arguments on one level, and on another, the theories, themes and results out of which they were constructed. We chose this method in part because it allowed us to accommodate a relatively large number of articles for synthesis and construct the “story” that emerged from them. This story of the body of literature is depicted in the themes that form the results of this article, and the overall conceptual ideas applied in the discussion.

### Data sources and search strategy

The WHO maternal morbidity definition is accompanied by a comprehensive matrix of conditions, symptoms, signs and management [7] which clarifies what it includes and which we used during the searching and screening process.

**Fig 1:**
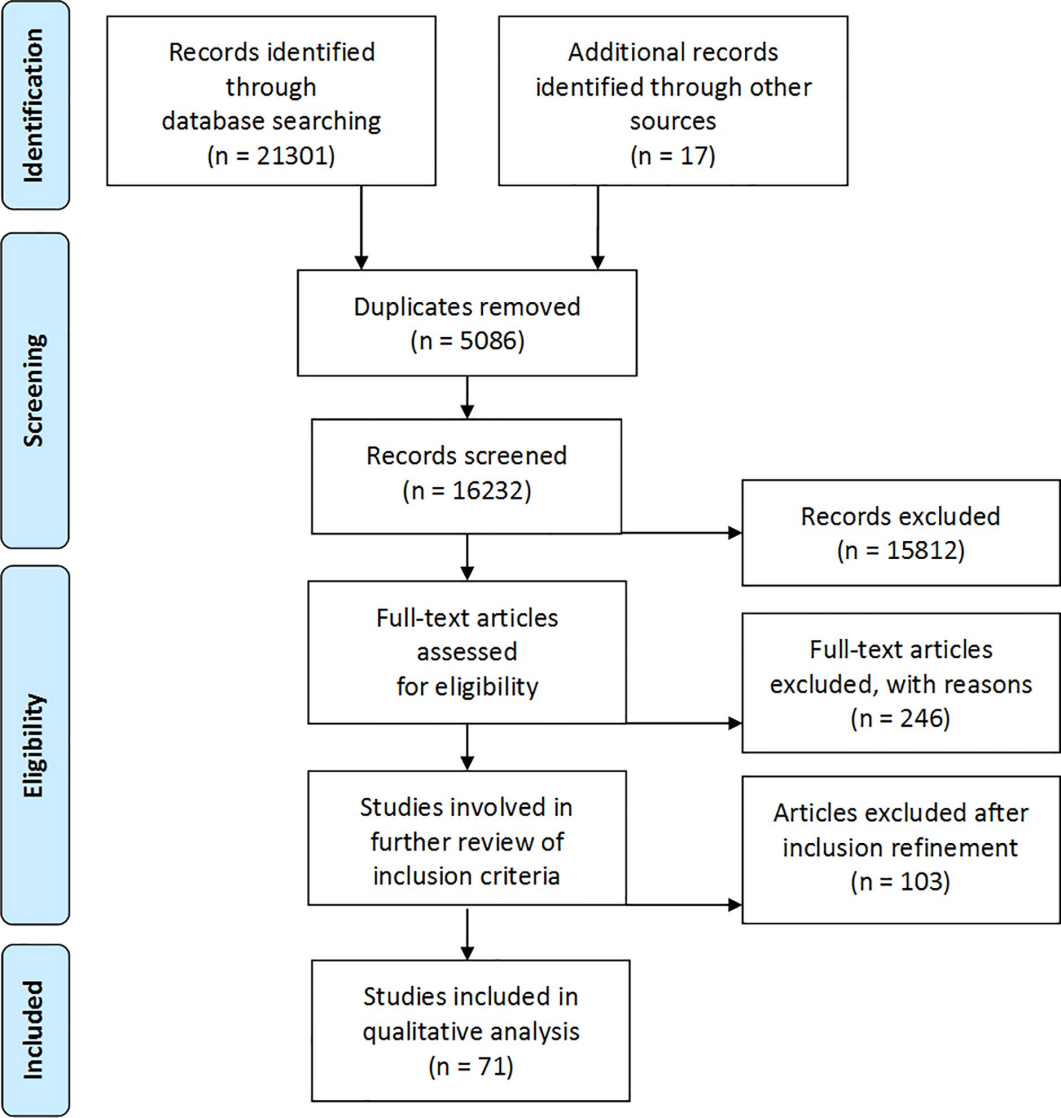
Qualitative literature on maternal morbidity screening process (PRISMA flow diagram).

We sought articles published between January 1998 and January 2018 in eight electronic databases: Medline, Popline, CINAHL, EMBASE, SCIELO, Anthropology Index Online, Anthrosource, and LILACS (Latin American and Caribbean Health Science Literature). We customised search strategies for each database using subject headings, as there was considerable variance in searching interfaces among the databases. The search strategy employed MeSH and free-text terms for maternal morbidity based on a partner review strategy undertaken on the quantitative measurement of maternal morbidity and health functioning [22]. For example, for the Medline and Embase databases, using proximity operators and appropriate truncation characters, we searched using terms for variations on maternal status (i.e. obstetric, pregnancy, labor, postpartum) and explicit morbidities (i.e. ectopic pregnancy, hemorrhage, prolapse, and incontinence) in addition to general terms for morbidities (i.e. complications, health, well-being, impairment, and morbidity). We also included country qualifiers: lower, lower-middle, and middle-income countries were identified using the World Bank classification [23] and included in the search strategy, though only lower and lower-middle were selected and analyzed for the review. We adapted Medline’s qualifier for qualitative studies to restrict the methodological approaches included and allow for different types of qualitative methods such as interviews, focus groups, ethnographic, participant observation and narrative data collection. (Please see S1 Appendix 1 for the Medline and Embase search strategy.) In order to capture the widest range of articles on our topic in the databases with simpler search engines, such as the anthropological ones, we worked broadly with variations of the terms maternal, pregnancy, obstetric, health, morbidity, well-being, illness and sickness, and did not restrict by method or country. (Please see Fig 1 for details outlined in the PRISMA diagram).

#### Inclusion and exclusion criteria

We assessed studies published between 1998 and January 2018 which used qualitative methods for data collection and analysis. The 1998 start date was selected to include research undertaken in the years just preceding the establishment of the MDGs in 2000. Articles included stand-alone studies or discrete parts of larger mixed-method studies if the qualitative component featured in the description and analysis in the paper. We included papers whose objective was to understand women’s experiences of maternal morbidity, but excluded if they focused on: only women’s knowledge of signs and symptoms of illness; hypothetical situations; perceptions of quality of care; and barriers to accessing maternity care. We further excluded studies not carried out in low or lower-middle income countries; studies in which subjects were not pregnant women or not women who had been pregnant; and studies in which the results, analysis and/or discussion did not relate to maternal morbidity or well-being. Maternal morbidity could be diagnosed by clinical or laboratory examination, self-reported, or defined by the investigators. Long-term morbidity and sequelae were included. We excluded editorials and literature reviews. Monographs were excluded, however, even when searching the databases, no monographs appeared that matched our other inclusion criteria.

### Selection and data extraction

Three authors (ILL, AG, VF) screened titles and abstracts of an initial sample of 100 papers to determine inclusion and exclusion criteria; ILL and ML then screened all titles and abstracts, and VF helped reach consensus where uncertainty arose. Data extraction was conducted by ILL, AG and RR who read the articles and completed an extraction table detailing the following content: location of study, study dates, publication dates, study design, the study population, sampling, maternal morbidity, data collection methods, funders, author profiles, analytical themes, findings, recommendations made.

### Analysis and synthesis

In the first step, we synthesized the authors’ descriptions of researched morbidities into illness-groups. Then, using the extraction table, we carefully read through the analytical themes and findings of each article, and classified them into iterative themes. We included data from the articles in our analysis that may not have featured in the authors’ main messages, but were building blocks for the article’s argument. For example, a study that took fistula as its morbidity starting point may have referenced the role of husbands (which emerged as one of our core themes) in women’s experience, just as did one for which malaria was the morbidity, without either article focusing on this as their main argument. In the next step, we developed a preliminary synthesis that was descriptive of the results that were presented in the studies. After this, we explored the relationships between these themes: re-reading the original articles, and distilling the many themes into higher level constructs, out of which emerged the ten topics that formed the basis of our thematic results. Finally, our analysis involved describing these ten topics through writing up the paper, which itself forms a part of the analysis.

#### Quality assessment

We completed [24] quality reviews for the first ten papers before agreeing that these criteria were not necessarily appropriate given the objective of our paper. A formal quality assessment according to outside criteria was peripheral for the results of our review, as the variance in approach, methods, and analysis of all included papers was critical for our analysis. The CASP method involves rating individual components of the research as written up (data collection methods, ethics, conceptual clarity, etc.) and an overall assessment of whether the quality of the paper is good, fair or poor. Our aim was to understand how and which topics in women’s experiences of maternal morbidity were given attention and which themes and insights emerged for the researchers. We assessed and weighed the quality of the pieces during analysis when the higher-level core themes developed by evaluating the appropriateness of their study designs, application of methods and the results of their analysis. All articles appear in the descriptive tally of the review and in the thematic analysis, but some do not feature significantly in the thematic write-up of the results and discussion if their methods or analysis were considered thin. In this sense, we reverted to a descriptive quality assessment, that is, a critical reading in light of the literature overall.

## Results

Our initial search identified 16,232 potentially relevant studies, in low, lower-middle, and middle income countries. When focusing on low and lower-middle countries, we retained 420 papers after screening titles and abstracts, 174 after full-text screening, resulting in 71 papers for inclusion (Fig 1 and S2 Appendix 2).

### Descriptive characteristics

#### Geographical distribution, years of publication, and maternal periods studied

Five of the six World Bank regions that at least partially include low or lower-middle-income countries were represented in the study papers, with only one paper from the Middle East or North Africa (Morocco). (Please see Table 1.) Sub-Saharan African countries most represented were Anglophone (Uganda (8), Ghana (6), and Tanzania (6)). Bangladesh (8) and India (6) led in papers from South Asia, meaning that just under half of the studies took place in five countries, while 23 countries were represented overall (out of a total of 82 countries in these two income categories) [23]. Our wider initial literature search showed that many papers in middle-income countries addressed maternal morbidity, however low-income countries appeared only 42 times, and lower-middle-income countries 30 times (more than 71 represented due to papers that studied multiple countries).

**Table 1:** Geographical distribution, years of publication, income classification of countries (*total adds up to more than 71 as one article covered countries classified in two income groups).

	Papers	Countries represented	Total N = 71
**Region**			
**Sub-Saharan Africa**			
West & Central	(Ahorlu et al., 2007)(Alio et al., 2011)(Bass et al., 2008)(Dako-Gyeke et al., 2013)(De Allegri et al., 2007)(Desalliers et al., 2017)(Mensah et al., 2017)(Mwini-Nyaledzigbor et al., 2013) (Murray et al., 2012)(Soderback et al., 2012) (Storeng et al., 2010)(Sullivan et al., 2016) (van der Sijpt, 2010)(van der Sijpt, 2014) (van der Sijpt & Notermans, 2010)	Ghana, Niger, Burkina Faso, Liberia, DRC, Cameroon	15
East & Southern	(Ashaba et al., 2017a)(Ashaba et al., 2017b)(Changole et al., 2017)(Chapman, 2006)(Dennis et al., 2016)(Donnelly et al., 2015)(Drew et al., 2016)(El Ayadi et al., 2017)(Hanlon et al., 2009)(Hannig, 2015)(Kaaya et al., 2010)(Kabakyenga et al., 2011)(Kay et al., 2015)(Kaye et al., 2014b)(Kaye et al., 2014a)(Khisa & Nyamongo, 2012)(Khisa et al., 2017a)(Khisa et al., 2017b)(Mbonye et al., 2006)(Mselle et al., 2011)(Mselle & Kohi, 2015)(Mukwenda et al., 2017)(Muleta et al., 2008)(Mwanri & Gatwiri, 2017)(Rosario et al., 2017)(Stewart et al., 2015)(Turan et al., 2007)(Yeakey et al., 2009)(Yeakey et al., 2011)	Eritrea, Ethiopia, Tanzania, Uganda, Kenya, Malawi, Mozambique, Rwanda	29
**Middle East &** **North Africa**	(Assarag et al., 2015)	Morocco	1
**Latin America &** **the Caribbean**	(Berry, 2006)	Guatemala	1
**South Asia**	(Akhter et al., 2017)(Chatterjee & Fernandes, 2014)(Clarke et al., 2014)(Edhborg et al., 2015)(Head et al., 2011) (Iyengar et al., 2016)(Kalim et al., 2009) (Matsuyama & Moji, 2008) (Naved et al., 2012)(Rodrigues et al., 2003) (Sibley et al., 2007)(Sibley et al., 2009) (Ravindran et al., 2000) (Thippeswamy et al., 2015) (Uzma et al, 1999)	India, Nepal, Bangladesh	15
**East Asia & Pacific**	(Andajani-Sutjahjo et al., 2007)(MacLellan, 2010)(Montesanti, 2011) (Niemi et al., 2010)(White, 2002)	Cambodia, Vietnam, Indonesia	5
**Multiple low &** **lower-middle**	(Heller & Hannig, 2017)(Maulet et al., 2015)(Menaca et al., 2013)	Mali, Niger, Ghana, Ethiopia, Kenya, Malawi	3
**Multiple low,** **lower-middle & middle/upper**	(Horowitz et al., 2001)(Oates et al., 2004)	India, Uganda	2
**Income classification**			
**Low**			42*
**Lower-middle**			30*

A general increase in qualitative papers on maternal morbidity over time was observed over the twenty years of this review, with 0–2 papers per year between 1998 and 2005, six papers in 2010 and 2011, and a peak of thirteen papers in 2017.

#### Maternal stages covered

For the most part, the included studies did not restrict themselves to the traditional maternal mortality definition of 42 days for postpartum. At least 45 papers considered longer postpartum periods for analysis. (Table 2) Many of the papers studying the pregnancy period focused on mental health.

**Table 2:** Maternal stages studied.

Timing of study focus	Total: 71
Antepartum	9
Antepartum & labour	1
Antepartum & postpartum	8
Postpartum (< 6 weeks)	3
Postpartum (> 6 weeks)	24
Postpartum (both < = 6 weeks and > 6 weeks)	21
Labour, postpartum <> 6 weeks	5

#### Morbidities studied

The three areas of morbidities receiving the most attention were 1) the consequences of obstetric fistula (24 studies, with 15 of these published since 2015); 2) mental health issues in either or both pregnancy and postpartum with 16 papers (postpartum depression with six, depression in pregnancy and postpartum with four, anxiety or distress with five, and one paper on postpartum psychosis); and 3) complications at labour and delivery with seven studies focusing on near-miss (referring to women who nearly died but survived a complication in pregnancy, childbirth or postpartum [25]), and five on other complications. (Table 3) Three papers dealt with the inability to have children, either through the exploration of infertility or pregnancy loss. Clinically, only secondary infertility might be a maternal morbidity or a maternal morbidity sequelae, but these studies did not distinguish between primary and secondary infertility and we have included them, especially as sub-fertility is a key element of maternal history identified in the Chou et al matrix [7]. Seven studies examined women’s experiences of a range of maternal morbidities grouped together. Many of the morbidities researched overlapped. For example, papers that focused on obstetric fistula referenced ensuing mental health issues, however, as the physical ailment was the point of departure for the studies we classified them as such. Two papers focused on depression amongst pregnant or postpartum women with HIV, and were classified in this table under mental health.

**Table 3:** Morbidities studied in papers, listed by primary morbidity, and countries of study. N = 71.

Morbidity	Number of papers	Countries in which research undertaken
Obstetric fistula	24	Eritrea, Ethiopia, Malawi, Niger, Tanzania, Kenya, Liberia, Ghana, Rwanda, Mali, Burkina Faso
Near miss (any/all)	7	Bangladesh, Burkina Faso, Uganda, Morocco, Tanzania
Postpartum depression	6	India, Uganda, Indonesia, DRC, Ethiopia
General pregnancy & postpartum health	6	Cambodia, Mozambique, Bangladesh
Labour & delivery complications	5	Guatemala, Nepal, Bangladesh, Uganda, Ghana
Anxiety, distress	5	Cambodia, Tanzania, Ghana, Nepal, Bangladesh
Depression in pregnancy & postpartum	4	Vietnam, Malawi, Uganda
General postpartum health only	3	Bangladesh, India
Malaria	3	Uganda, Ghana
Pregnancy loss & infertility	3	Cameroon
Postpartum psychosis	1	India
Mastitis	1	Burkina Faso
Uterine prolapse	1	India
Anemia	1	India
Gestational diabetes mellitus (GDM)	1	Ghana

#### Data collection methods

Well over half the studies (43) based their results on semi-structured or in-depth interviews. Due to discrepancies and ambiguity in the characteristics of semi-structured and in-depth interviews presented in the papers, we have categorized these together. Three studies employed solely focus group discussions (FGDs), and ten used interviews and FGDs. In addition to interviews and FGDs, a further four studies employed targeted qualitative methods such as free-listing, mapping, and attribute ranking. Eleven studies involved a more ethnographic approach in both methodology and analysis, meaning that participant observation formed a central part of data collection, and a theoretical approach guided fieldwork and writing-up.

### Thematic analysis

**Fig 2:**
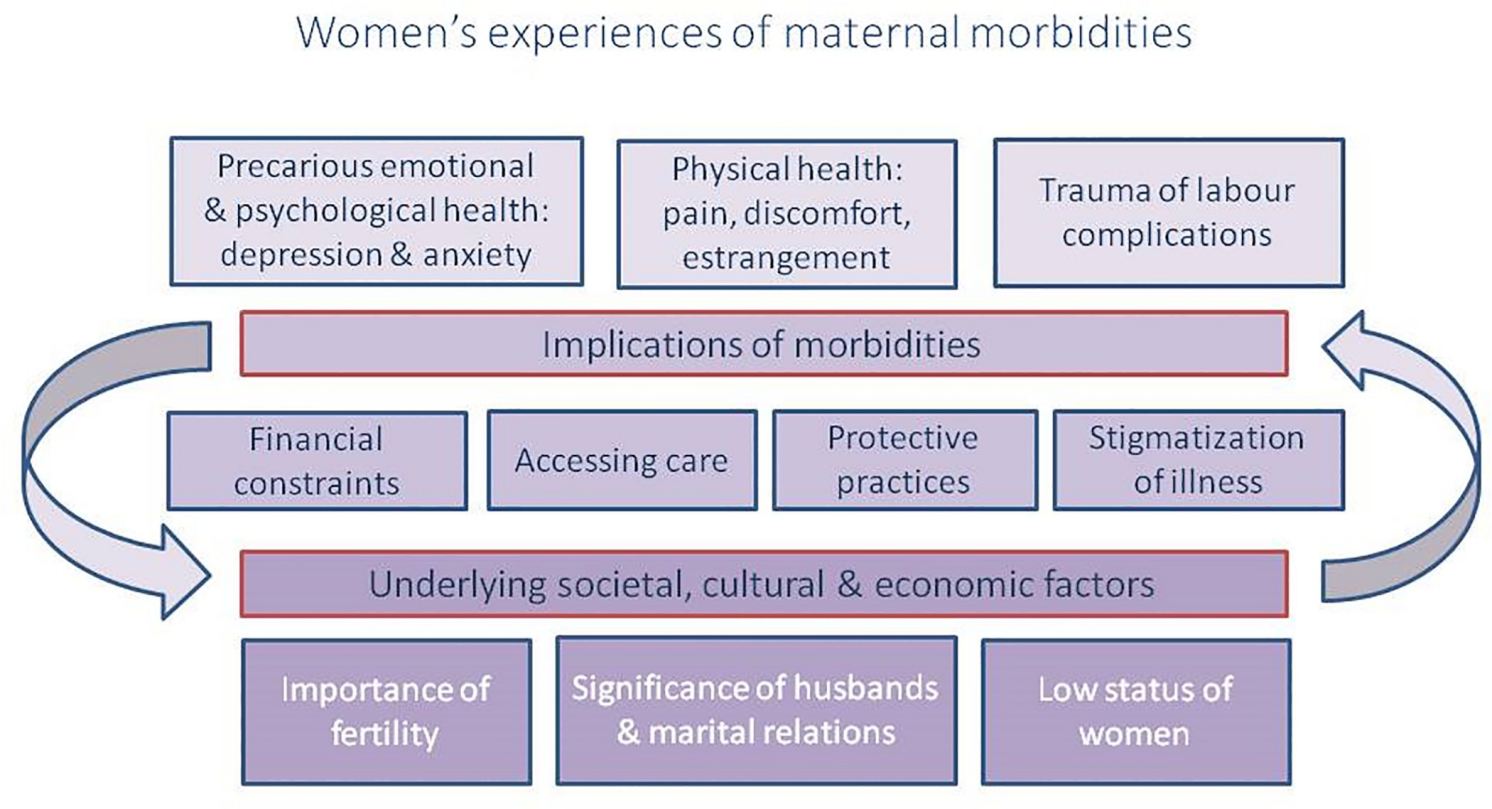
Major themes in qualitative literature on maternal morbidity.

While many themes emerged that were specific to individual papers or small subgroups, the overarching themes emerging from the retained articles refer to underlying contextual factors (including societal, cultural and economic factors) into which the morbidities manifest and the implications of the morbidities on women’s lives. There is interplay between the context and the experience of maternal morbidity, and the boundaries between the themes overlap with shared elements and repercussions. (Fig 2) Below we describe each theme, giving examples from the literature that are not exhaustive of all articles speaking to that particular theme, but which offer insight into its expression across the papers.

#### Underlying social, cultural and economic factors

1 Low status of women

Generally, papers focused on women with limited schooling and resources and the findings of this review are a reflection of that. Permeating studies was women’s lower status vis-à-vis men, even when they are healthy, and their subordinate positions in families and communities that put them at a disadvantage in terms of education, equality in their relationships, autonomy, decision-making, and access to resources to allow them to make informed health care decisions and follow through on them.

When maternal morbidities come into play, matters can become compounded. Chatterjee et al studied perceptions and practices related to anaemia among pregnant women in Mumbai and showed how “anaemia is almost symbolic of deeply entrenched gender inequalities in India that manifest on the plate of food served to girls and women” ([26] p. e62). Women in the communities she studied typically first served men at mealtimes and then themselves, with the consequence that their meals were less nutritious, particularly given the scarcity of nutrient-rich foods and diets constrained by cultural and religious practices (even when women were pregnant). Women understood that anaemia related to nutrition, but not which foods prevented it. “Anaemia does not just stand at the intersection of health and nutrition; it also stands at the intersection of culture and gender. From a socio-cultural point of view, we are not just dealing with women’s knowledge gaps about anaemia and nutrition, but also the low status of women in the Indian household.” The authors continue with their assessment of the origins of this health challenge beyond iron intake: “We have to address those socio-cultural injuries and injustices that start at birth of the girl-child and continue to influence women’s treatment right up to their old age” ([26] p.e62).

In a detailed ethnographic paper on women’s reproductive lives in central Mozambique, Chapman [27] links pregnancy risks with social inequalities through the analysis of the complex relationships between the material and physical worlds. She describes how women’s reproductive pathways are as much social as they are biological and include practices that can place them at a disadvantage. In her examples, economic marginalization keeps women distrustful and in competition with other women–missing opportunities for mutual support. Dependence on a spouse’s earnings means that they feel pressure to procreate through their fertile years. Women are vulnerable, which she defines by quoting Mark Nichter, as “the actual feeling of susceptibility to illness or misfortune. It is a state of weakness, fear and worry” (Nichter as quoted in ([27] p. 495)). This vulnerability plays out on women’s abilities to care for their pregnancies, placing them at risk for obstetric loss and complications (in addition to challenging lives), and she calls for public health’s conceptualization of risk to include the structural violence that “inform(s) perceptions and limits choices” (ibid p. 509).

These examples of how women’s status is addressed in this body of work form a critical backdrop to the descriptions and analyses of women’s experiences of maternal morbidities in this review. Hardly a study was presented without underlining contextual variances on this theme, and the compromised position from which women exercise their health rights. At the same time, it is important to recognize that an act of subordination in one context will not be perceived as such in another, and that women’s subordinate position in their families in some settings does not necessarily reflect their own lack of agency, but rather that of society’s role-setting for all actors, not just women.

2 Significance of husbands and marital relations

No paper focused exclusively on marital relationships, or endeavoured to understand the problems with intimate partner violence or other types of abuse exacerbated by or aggravating the maternal periods. However, it was clear that husbands were critical to pregnancy and post-natal experiences (though papers generally did not elucidate the structures and constraints that impacted men and their decision-making). Whether husbands or other types of partners, these men were referenced as playing roles in determining access to health care (for example [28,29]); financial dependence [30–32]; fear of abandonment or the loss of a stable relationship [33,34]; and absence or presence of a support system [35–37].

These issues were found across all regions and the range of morbidities, but featured strikingly in papers focusing on obstetric fistula and mental health (both of which are chronic morbidities and our two largest categories of papers). Much of the stress linked to husbands was emotional and related to uncertainty surrounding norms and (spoken or unspoken) agreements in personal relationships. Naved et al, when describing women with long-term maternal morbidities in Bangladesh, detailed women suffering “emotional abuse relating to [their] inability to satisfy husbands’ sexual needs” ([38] p. 187) due to consequences from fistula, uterine prolapse or stress incontinence. Authors also attributed mental distress during the pregnancy and postpartum periods to feeling troubled and despairing about husbands who were not supportive or understanding [39,40] and fear of husbands being unfaithful [41], which was even seen as a threat to a healthy pregnancy.

The families of husbands also featured heavily, mainly in pressures to conform to their requirements and expectations [42–44]. In particular, mothers-in-law were attributed these characteristics, and featured in articles across South and East Asia, and West, East and Southern Africa as being controlling, influential for decision making, and leading to increased stress for unwell daughters-in-law both during and after pregnancy. The wide geographical range of this theme indicates that though the context of fertility and a woman’s place in the family is regionally specific, many of the same overarching issues are played out.

Although instances were highlighted in which husbands played supporting roles that had positive outcomes [45–48], the articles’ authors emphasize that a primary worry for women is her relationship to her husband and his role in her health and care, signifying either a point of focus on the part of the researchers or women (or both groups).

3 Importance of fertility

Women’s fertility features centrally to their public and private identities in all papers. Not only did papers on pregnancy loss and infertility illustrate this, but others also revealed how women were frequently in large part valued for their reproductive capacities. Examples include women with “chronic maternal disabilities’” (defined by the author as impairments resulting from severe acute complications) low status in the community linked to their inability to bear more children (in Bangladesh [38]) and women’s fear of not being able to produce living babies for/with their husbands creating anxiety and distress (in Liberia [49]). Mselle et al [50] note that among their sample of women with fistula in Tanzania, those who already had living children before the fistula recounted more positive experiences of living with the morbidity than those who did not.

Van der Sjipt [51,52] and van der Sjipt and Notermans [53] provide analytical detail on perceptions and practices surrounding pregnancy loss in Cameroon and how the physical and the social body are inextricably linked. In her 2014 paper elaborating on the narrative of one woman’s journey with pregnancy, miscarriage, separation, and struggles with status and employment, van der Sjipt analyzes the role of fertility beyond its medical attributes in a way that resonates across other papers. She builds on the concept *vital conjunctures*, a term used by Johnson-Hanks in her ethnography on unintentional motherhood in Cameroon [54], which refers to “socially structured zones of possibility that emerge around specific periods of potential transformation in a life” ([54] p. 22). In van der Sjipt’s analysis of the weight and status of fertility, she writes: “The *social body* [our emphasis], without discarding the materiality of physical experiences, draws attention to its inherent social aspects as well; *vital conjunctures* offer a space to explore how both physical needs or constraints and socially constructed wishes, options or restrictions influence reproductive behaviour. Through this sociocentric approach, pregnancy losses emerge as heterogeneous rather than homogeneous, and situational rather than universal events” ([51] p. 1779).

#### Implications of women’s experiences of morbidities

4 Precarious emotional and psychological health: depression and anxiety

Sixteen papers specifically focused on mental health–not only depression, but also feelings of unease, anxiety and worry. Of these, seven based their findings on research in Asia, and nine in East or Central Africa. Most other papers also included the emotional and psychological experiences of women in their analysis of how they lived their illnesses, as expressed across the ten themes elaborated in this results section. In fact, many scholars recommended complementary, holistic treatment approaches across the range of morbidities–from HIV to GDM, to near miss complications and fistula–that included improved consideration of women’s psychological responses and their coping with illness and recovery [34,35,55–60].

Some authors used qualitative tools to explore the universality of diagnostic criteria. Many mental health papers examined how women manifest symptoms of psychological distress and mental illness which the international psychological and psychiatric community recognize through actions and emotions. Several studies implemented one or more quantitative psychological tools to validate the existence of depression or postpartum depression in different settings. For example, Bass et al [61] aimed to explore whether postpartum depression existed among Kinshasa mothers by using interviews and two depression-screening tools. The authors found that the local term for mental health morbidity defined it as “thinking too much” ([61] p. 1541). The results from Oates’ multi-country study support the existence of “the universality of a morbid state of unhappiness following childbirth” ([43] p. s10), though not all study participants referred to or recognised this state as post-natal depression or “baby blues”. They also determined that solutions to support women’s mental health had to be tailored to context-specific circumstances. Along the same vein, these authors illustrated results found across all the papers in this review, in that “the contributors to happiness and unhappiness were not necessarily the converse of each other” ([43] p. s12). For example, distress surrounding husbands contributed to women feeling unhappy after childbirth, but positive experiences with husbands were in turn cited as contributing to women’s postpartum well-being.

Similarly, Niemi et al [62] set out to understand whether in Vietnam depression was associated with the postpartum period, or if this was a “western construct”. They found that the women and health workers interviewed overwhelmingly considered social factors as the cause of general depression, including post-natal depression, which was not recognized as a separate category among their informants. The triggers were problematic relationships, lack of support, vulnerability, and economic problems–echoing what we have heard from other studies. Notably, their investigation also found that some mental illnesses were attributed locally as “thinking illnesses” because there was less stigma associated with this label than with a mental illness attributed to biomedical causes. This finding underlines the complexity of using static, predefined categories to measure, classify and understand the meaning of mental illnesses in diverse settings. It also raises broader questions for the possibility of reliably describing morbidities comparatively.

The impact of a mother’s psychological health on the health of her children was cited in at least two studies [61,62] as a rationale for conducting qualitative research on maternal mental health.

5 Protective practices

A discourse throughout the studies concerned “cultural” adaptations to pregnancy and childbirth. In childbirth, individuals and societies try to respond to and accommodate the associated physiological and social changes, and to make sense of aberrations and complications. Many articles described what we call here “protective practices”, which are measures ostensibly designed to make the pregnancy and postpartum periods easier for women, such as taboos against postpartum intercourse [63], women’s confinement at home for a number of days after childbirth [64] and the adoption of specific postpartum diets [35]. Studies revealed that while these practices were intended to improve a pregnant woman’s well-being, they frequently actually responded to the ideals of a woman’s entourage or the desire to create culturally acceptable scenarios.

Protective practices were regularly analyzed with reference to mental health. The act of bringing life into the world–childbirth–can involve cultural rites of passage to protect women from perceived risks, such as postpartum depression or infection, but nevertheless, some responses could actually “increase mothers’ vulnerability” ([35] p. 1803). Hanlon et al’s study of postnatal mental distress in Ethiopia maps out the phenomenon of intended and unintended consequences of protective practices [65]. In their study setting, participants recognized postnatal confinement (generally between 40 days and 3 months) as a time to be “savored” but also acknowledged that for some women it could be a time of feeling excluded, potentially exacerbating pre-existing difficulties. In addition, traditional postpartum practices designed to shield the baby from spiritual dangers could end up being taxing for women and their communities when they were unable to meet expectations. The authors pointed to societal transitions and change as influencing women’s ability to adhere to traditions. “Participants identified the mismatch between expectation and actuality as a potent stressor for some postnatal women, leading to manifestations of mental distress” ([65] p. 1216).

Maclellan et al [66] detail the custom amongst their Cambodian informants of not discussing childbirth or sharing information about pregnancy with pregnant women–even between mothers and daughters–from conception through to the postpartum period. This created anxiety even though it was devised, or billed, as being a protective measure to relax the expectant mother about what lay ahead of her. Maclellan states, “The cultural belief that withholding information […] reduces an individuals’ anxiety is clearly challenged by the insight of these women, and may actually be related to a lack of knowledge on the part of the information giver rather than a concern for the level of anxiety in the subject” ([66] p. 741).

These papers demonstrated that socio-cultural norms and “helpful” restrictions placed on women can benefit women and sometimes also hurt women, even when their stated intention was to be positive. In certain settings, women who deviated from expected practices ran into greater difficulties with their recovery, or aggravated their original problems. Some scholars deemed knowledge of these practices critical to the development of interventions and communication campaigns aimed at benefitting new mothers [67].

6 Physical health: pain, discomfort, estrangement

A common theme across these papers is the intimate entanglement of pain and discomfort in the experience of morbidities, and the subsequent impact it can have on the rest of women’s lives. In the studies on maternal depression, fatigue and pain from delivery complications were frequently physical symptoms that accompanied the emotional or psychological responses [35,68].

For example, Mselle et al [69] in their study on women with severe birth injuries in Tanzania (including fistula), link women’s experience of pain with their loss of bodily control as they are unable to perform in their communities as they would normally. For some women with maternal morbidities, it was not only the stigmatization of their illness that made them feel removed from society, but also the discomfort they felt. This was particularly evident in the articles on fistula, where women narrated sore, aching bodies and skin that accompanied the wetness they could not control [70,71].

Not only the illness itself could be hurtful, but the process of undergoing treatment and recovery could be too. De Allegri et al [72] in Nouna, Burkina Faso, describe painful traditional medical practices exercised to prevent or reduce mastitis, making caring for herself and her baby’s health a more demanding, engaging process for women during an already compromised period. These and other papers are examples of the expectations placed on women to maintain roles through their pain, and to the personal feelings of loss and hurt that accompany the repercussions of pain.

7 Financial constraints: economic burdens of maternal morbidities

Linkages between maternal morbidities and personal economic burdens fell across three areas: the consequences of a lack of funds to provide and seek care; the encumbering costs of health care; and the implications of illness on earning capacity. While this review did not specifically focus on access-to-care barriers, in many instances it was impossible to divorce the inability to obtain care or vacillation in decision-making surrounding health care from the experience of morbidity.

Personal finances acted as a barrier to care-seeking for women’s morbidities, which at times then exacerbated the condition and increased the chance for complications [29,30,42,73]. Financial hardship that was incurred through heavy expenses for treatment produced uncertainty, stigmatization, pain, worry and interrupted treatment [34,74,75], though there were also tales of financial support from the community and family [76,77]. Other papers detailed the repercussions of not being able to continue working due to ill health, disability resulting from pregnancy or childbirth, or stigma associated with the morbidity [38,55,78–81]. Kay et al [82] described the frustration and disappointment women felt due to no longer being able to provide for their loved ones in their sample of patients seeking surgical treatment for fistula in Rwanda.

The losses were not simply about earning an income, but also about missed opportunities [83,84]. Some informants and scholars recommended that financial support in the form of loans, business development, or direct coverage of medical expenses be given to women coping with maternal morbidities ([60,85].

8 Trauma of severe labour complications

Several studies highlight the bodily trauma of obstetric complications and the long lasting economic, social and physical effects these can have. Many featured complicated deliveries, including those in which women narrowly escaped death (near-miss), as their starting point and aimed to understand the impact of these morbidities. Murray et al [84] followed Burkinabe women who experienced near-miss deliveries in a longitudinal study over four years and show how from the challenge of surviving the delivery through to the recovery and reconstitution of life, complicated childbirth can have lasting repercussions. “The ‘near-miss’ event was a ‘turning point’ that significantly altered their perceptions or life course” ([84] p. 2458). Women who had access to a variety of financial and social resources had better chances of moving beyond their obstetric trauma (see also Storeng et al [74] from the same study).

These events also shaped the experience of subsequent maternal morbidities, for example emotional stress and difficulty adjusting to motherhood and the post-pregnancy period [63]. Papers document how the resulting “loss” from traumatic births extended beyond the physical to social and financial loss. Some authors underlined the tangible support given women by friends and family in the aftermath of difficult labor [86]. Various authors made calls to improve counselling and support services for women who experience near-miss or other catastrophic childbirth [55,80].

9 Stigmatization of illness

A theme in many papers was the stigma associated with maternal morbidities, which played out in terms of ostracization, changes in social status, and had effects on women’s psychological, economic and social well-being. Where in some instances women received support from their networks, they also battled the judgment and expectations of the wider community. Many then tried to hide their conditions to avoid stigmatization, which led to diminished care seeking for help that could have alleviated their morbidities [56,62]. A fear of not being understood or being perceived to be weak or defective hampered women with postpartum depression or other mental health issues from seeking care [65,68]. Women were also blamed for bringing on their illnesses (even by themselves) [39,49], which led to further stigma. Faith in God was highlighted as giving strength to cope with the stigma of illness [60] as well as making it through illness and recovery [57,87].

In particular, papers on fistula highlighted the stigmatization that women felt with their condition, often emphasizing the effect of negative social responses also on their husbands. A fistula patient’s husband in Tanzania said, “‘ …the community started laughing at me saying that it is better I divorced my wife because she was leaking urine and that she cannot deliver again’” ([69] p. 56). A 42-year-old woman of high socio-economic status with stress incontinence after childbirth also drew attention to the response to her illness: “The people in the community who know about my condition laugh at me. They treat me with disgust (ghinna-minna). I avoid them” [38].

Notably, some of the more recent papers respond to the historically dominant narrative describing the heavy stigma women with obstetric fistula face by depicting contrasting experiences [47,88,89]. For example, in their study in Tanzania, Dennis et al [47] emphasize the helpful roles that the wider family plays for many women, even when husbands are not supportive. They also found that problems with husbands frequently predated the fistula, and were not the result of the morbidity alone. Similarly, Heller and Hannig [89] note that women often remain integrated in social networks and obligations for years even with their fistula, and describe how these women’s trajectories are not always the same as those that have been popularized, where women are abandoned and surgery offers a straightforward solution.

10 Accessing care

In the above sections, we referenced multiple barriers to accessing care. Men controlled either decision-making or finances, or both; measures that were meant to protect women ended up limiting them in their choices; and poverty and lack of education meant that women struggled to reach the care they sometimes desired. Access can refer to both physical contact with care as well as decision-making in the sense of interpretations of illnesses, diagnoses, and their necessitation of care. Some studies advanced reflections on this area by trying to look beyond the tangible boundaries to accessing care and instead conceptualize other obstacles, such as the recognition of symptoms, their interpretation, and acting upon them (for example [90–92]).

To illustrate this complexity, we can take the example of intrapartum bleeding and how varying understandings of blood loss can influence health seeking. Sibley et al [93] and White [94] describe how even when there was an agreement among carers that excessive bleeding required attention, the ambiguity in terms of what constituted “excess” meant that care seeking could be compromised. Khmer women believe their bodies need to expel “bad blood” before bleeding is considered excessive [94]. Kalim et al [95] show that in their study site in Bangladesh, when excessive bleeding is recognised, next steps still depend on elderly family members to mobilise care seeking and may result in delays. Further difficulties included the challenges of measuring blood loss by both lay and professionals, and Matsuyama et al [28] describe the symbolism of blood, and how embedded the meaning of bleeding is in different communities which in turn impacts decision-making. Research shows that not only the (mis)interpretation of signs in pregnancy can lead to the development of maternal morbidities, but also the (mis)identification of diseases (such as malaria [31]).

As mentioned previously, a number of studies detailed the harmful repercussions of women’s desire to conceal their morbidities from family members and the greater community–in particular the delays to care-seeking that this could bring about. Attempts at “passing” as not being ill could result in psychological disturbances and fatigue as well as the exacerbation of symptoms and further poor health [62,89,96].

## Discussion

Our review finds that there are structural factors that greatly influence women’s experiences of maternal morbidities, including their status, the importance of fertility, and the role of husbands and their families. These factors set the scene for the how the social, physical and mental effects of morbidities play a role in women’s lives. The overlaps between our themes suggest that there is a commonality in experiences. However, we also found gaps in qualitative research on how women live with maternal morbidities.

### Review of descriptive findings

#### Morbidities in focus

Three morbidity groups dominated the results: obstetric fistula; mental health including anxiety, postpartum depression and psychosis; and labour and delivery complications. Over one third of all papers, 24, focused on obstetric fistula, a morbidity with a comparatively low prevalence but difficult to treat and with serious consequences. All included papers on this topic were from Sub-Saharan Africa where there are an estimated 6,000 new presentations a year [97]. The fact that fistula has in recent years received a great deal of United Nations (UN), donor and media attention may account for this (or be partly a product of this publication effort), as advocates and funders working in this area sought to bolster their cause with scientific evidence.

Between the categories of anxiety, distress, psychosis and postpartum depression, sixteen papers addressed maternal mental health. While screening, we came across many papers that exclusively used quantitative scales to measure postpartum depression, and therefore excluded them from our review. A sizeable number of papers focused on anxiety and distress without trying to fully medicalize or quantify the phenomenon using scales and diagnoses, instead focusing on feelings of worry, unease, distress and anxiety. These emotions were self-identified but fit the definition of a morbidity according to the women affected. Even in this qualitative sample, the gaze of most researchers was on postnatal depression (six papers exclusively, ten all together), signifying the attention this clinical category receives while other maternal mental health issues may be as concerning from the perspective of women living in these communities.

Twelve papers looked at the complications that occurred during labor and delivery, either near miss or other less severe medical problems. Given that the leading causes of maternal mortality are hemorrhage, hypertensive disorders and sepsis (for a combined total of 51.8% of deaths) [98], it seems that surviving women’s experiences of these complications are comparatively being left out of the research agenda. Papers that focus on women’s knowledge of signs and symptoms from a programmatic perspective–excluded from our review–do address these potential precursors to maternal mortality but are mechanistic in their approach to morbidity and access to services.

#### Geographical distribution

The majority of studies included were conducted in Sub-Saharan Africa and South and East Asia. While there are 41 countries in the ‘Latin America and Caribbean’ region, only five of its countries meet the low- and lower-middle income inclusion criteria. While screening (in which papers from middle-income countries were included), and while undertaking a related review [99], we encountered many more articles that matched our thematic objectives. This experience suggested that country classifications are not necessarily a representative category for understanding the issues that affect women in low-income settings, as there are pockets of disadvantaged communities in richer countries. Poverty, the status of women, access to information and health care, and many of the other themes encountered in our papers are themes not unique to low GDP countries.

#### Research methodology and quality

We noted an increase in papers on women’s experiences of maternal morbidity over the last fifteen years. While this could be indicative of greater attention paid to qualitative research on this topic, it also corresponds with a general increase in publications [100], the proliferation of open-access journals [101], and possibly a less stringent peer-review process for many of these [102]. We found a lack of papers that contribute deeper layers of understanding to the topic rather than replicating and disseminating evidence already generated, or not undergoing rigorous data collection and analysis processes to arrive at the findings. The article word limit of most journals (3500–6000 words) also imposes restrictions on the complexity authors can convey in their papers.

Over half the papers relied solely on one-off interviews with women and FGDs to investigate women’s experiences instead of triangulating their results with participant observation, carrying out repeat interviews, or including members of women’s entourages in their sample. It is well documented that what respondents say they do can be different from what they actually do and how they really feel [103], and that a topic with such an intimate objective may not lend itself to be studied best through single encounters. Asking direct questions leads to direct, and hence limited, replies, so that the depth and nuances of experience are neither expressed nor captured in straightforward data collection methods. In addition, fieldwork issues such as researchers mastering the language of their informants and employing assistants for data collection rather than author-researchers carrying out fieldwork themselves (among other issues) create distance between the researcher and the field of study, often leading to the development of less complex research questions and more superficial analyses of data.

#### Thematic analysis

The ten themes that are woven throughout this body of work underscore the interconnectedness of maternal experiences across countries, time, and morbidities. Three concepts can help us better recognize the threads that tie these experiences together and make sense of how maternal morbidities are lived: capital (social and bodily), biographical disruption and syndemics.

#### Social and bodily capital

The emphasis on a woman’s “responsibility” to produce healthy children (and herself remain healthy in the process); the impact her well-being has on her economic future–through the strength of her marital relationship and her ability to work effectively; and the social consequences of her morbidity within her family and greater community all point to the importance of both social and bodily capital. Social capital, a concept solidified by Pierre Bourdieu [104] and popularized in the last two decades in relation to inequalities in health, refers to the resources surrounding networks that individuals and collectives have in order to function soundly [105]. The notion of *bodily capital* gives a name to, practically quantifies, a person’s worth in society based on what they can contribute. Especially for poor people with a lack of material or financial resources, the body’s ability to produce (not just reproductively, but productively) is critical for survival [84]. In maternal health, the consequence of these shifts in capital are not only personal but also reverberate around the community within which a woman functions, often resulting in having to navigate the stigma of both her disease and the role into which she has been forced.

#### Biographical disruption

These papers show that for many women maternal morbidities result in a true “biographical disruption” [106] that is difficult to recover from, even after the physical effects of an illness have passed. Michael Bury uses this term to describe the consequences of *chronic* illness on a person’s life, trajectory and well-being: expectations are re-examined, the structures of everyday life are overturned, an awareness of worldly suffering develops, and relationships are altered when the normal rules of reciprocity and mutual support can no longer be enacted ([106] p. 169). This concept is not only applicable to maternal morbidities that have a long duration, such as fistula and some mental health issues, but case studies from our selected papers suggest that it is also a useful theory when extended to other maternal morbidities. The fact that these are *maternal* morbidities emphasizes this–they interact intensively with a woman’s identity and role in society, and as childbearing is at once so personal yet at the same time so public, it is not surprising that its consequences go beyond physical elements. We see that the effects of these illnesses are retained, and the mark on a woman’s life persists, even after returning to pre-pregnancy physiology at the end of the “traditional” six-week postpartum period. Anita Hannig [107] and others show that these disruptions do not exclusively weaken women or lead to less fulfilling lives.

#### Re-envisioning syndemics

Syndemics, first developed by Merrill Singer in the 1980s when he drew attention to comorbidity, forms the idea that diseases are not solely biological–created and living in bodies–nor do they exist in a social vacuum [108]. Social factors like education, poverty, racism, sexism, and structural violence play roles in the extent of a disease’s course and the toll taken on human well-being beyond simply the manifestation of compromised health. His point of departure was transmittable diseases such as HIV, and he looked at the factors that could layer on top of one another to influence how a disease would spread and to whom. The narrative of this review syncs with his analysis of pregnancy in sub-Saharan Africa [109] and indicates that it is not just transmittable diseases that are prey to such trajectories, but that patterns are evident for the collection of illnesses and diseases that are maternal morbidities, as well.

Our above analysis shows the social underpinnings of depression and distress in the postpartum period, how stigma and shame can exacerbate illness through inhibiting health seeking or creating anxiety, and how family structures and cultural practices can influence how morbidities are enacted and experienced. We have seen the “interrelation between social, economic and health problems during pregnancy” ([30] p.11) and call for the consideration of maternal morbidity to go beyond any solely biomedical definition and consider the perspectives of those who are living it.

As such, interventions to tackle the above challenges in maternal morbidity will not necessarily be medical, but will require broader approaches tackling inequalities and disadvantages engrained into the structure of many societies. As one author writes: “Social interventions that aim to influence deeply held norms about women’s roles, sexuality, and reproductive health will require time and must target influential women and men in families and communities” ([73] p. 1166). It is important to acknowledge that the existence of certain prevalent constraining social norms does not necessarily indicate that women are in oppressed positions, as Mumtaz et al [110] point out in their critique of the limitations of equating women’s autonomy with improved life circumstances (including reproductive health) through their analysis of reproductive decision making in Pakistan. They argue that policies with limited understandings of the scope of women’s independence “are unlikely to lead to effective action because of the incongruity between notions of individualism inherent in the concept of autonomy and the socially embedded reality of people’s lives’” ([110] p. 1355). Keeping this in mind while considering the syndemics of maternal morbidities, the SDG’s focus on underlying contextual factors such as the elimination of poverty (SDG1), good health and well-being (SDG3), gender equality and empowered women and girls (SDG5), clean water (SDG6) and reduced inequalities (SDG10) are suitable starting points for responding to the social challenges of maternal morbidity.

### Implications of this review

In conducting this extensive review of published qualitative work, we went beyond the main objectives and findings of each individual paper to identify the messages of the body of work as a whole. Our results point to several implications for the conceptualization of maternal morbidity and for research and programming. We make the following recommendations for initiatives in the field of maternal morbidity.

Conceptualization of maternal morbidity:

- The categorization, investigation and responses to maternal morbidities are evolving on national and global levels. Maternal morbidity should not just be seen from a clinical vantage point, as women’s experiences of their own maternal health shape their quality of life and well-being. A broader, life-course, women-centred understanding of the conditions that occur during pregnancy, childbirth and after pregnancy will allow for patient-centred priorities and perspectives to guide programmatic efforts and research agendas. This perspective is in sync with the wider reflections of the WHO MMWG, who have developed a Maternal Morbidity Measurement Framework [13].

Implications for programs and research:

- Beyond biomedical origins, complex structural, economic, cultural, ideological and socio-political systemic contextual factors underpin risks and experiences of maternal morbidities. Women’s personal agency and autonomy go only so far in explaining the constraints with which they live, and wider societal institutionalized norms need to be understood and taken into account when developing prevention and treatment programs.
- It is essential that interventions seek equitable solutions that reach and improve the situations of marginalised pregnant or postpartum women who are often left out [111]. In particular, this review underlined the constraints faced by poorer pregnant women who become unwell, but other marginalizing factors include disability, ethnicity and limits to education, among others.
- The impact of the interventions on preventing or treating the morbidity and associated negative consequences should be rigorously and *comprehensively* assessed with quantitative and/or qualitative methods. Program evaluation should take into account the multifaceted aspects of how morbidities are experienced.
- Research on how women and families encounter, interpret and make decisions around morbidities which are understudied but major contributors to mortality (such as eclampsia, pre-eclampsia and sepsis) could improve their outcomes. These morbidities continue to be difficult to research as they are acute and rare events, and investigation of families’ knowledge of signs and symptoms is not sufficient. Our analysis suggests that in many cases interventions should target a woman’s wider family or network, and not her alone, given how intertwined they are. With husbands and their families (particularly mothers-in-law in some settings) playing integral roles in women’s experiences of their pregnancies and morbidities, context-specific research is necessary to learn how and to what degree they should be involved. The appropriateness of this approach must be established on a topic and individual level, as in some cases increased participation of the male partner may place women at risk or exacerbate a sense of dis-ease.
- Some conditions emerging or on the rise in low and lower-middle income countries, such as gestational diabetes [112], have not been studied adequately in these populations, and should receive more attention from the interdisciplinary research, policy and programming communities. Increasing pre-existing risk factors of maternal morbidities, such as obesity and previous c-sections, also warrant more attention.
- Qualitative data that is actionable also has a role, such as implementation research on women’s experiences with their maternal morbidities that can be directly shared with program managers.
- Finally, we call for the pursuit of high quality qualitative research on maternal morbidities by trained researchers that includes repeat interviews, participant observation and triangulation of sources where possible. In addition to building rapport and capturing nuance, this can allow for the investigation of how the experience of morbidity changes over time.

### Limitations and reflections on the systematic review process

A limitation of this review was the narrow spread of literature ultimately included to gain insight into women’s experiences of maternal morbidity. The exclusion of monographs, which could contain rich descriptions and analyses of women’s experiences, is a limitation. Health crosses through many frames of human life, not only the medical sciences and associated disciplines. The way these are referenced, labelled and defined can place a limit on accessing women’s experiences from a variety of perspectives. The humanities are indexed differently, and though efforts were made to capture works that approached health from the humanities, some of these contributions may not have been picked up, imposing a biomedical lens onto this subject when women’s reference points are not all biomedical. That said, these are the databases commonly referred to by those researching and acting in the health sciences, and the results may be indicative of the most typical evidence accessed and consulted in this field. Our aim of capturing and analysing 20 years of qualitative research meant that we were able to show a systematic overview of activity, but that our analysis had to accommodate a heterogeneous range of methods, study objectives, morbidities and regional variations and was not as in-depth as it could have been had it focused within more finite and comparable classification groups.

This review, as a contribution to the efforts of the MMWG, signals a “seat at the table” [113] for qualitative research in defining maternal morbidities. This review also questions the place for “context-insensitive summaries of ‘the evidence’” [114] and we call for further reflections on creative interdisciplinary approaches to respond to questions that can be found in the body of published research results.

## Conclusion

This systematic thematic analysis has consolidated and analysed the heterogeneous body of qualitative literature reporting on maternal morbidity as lived and perceived by women, though the filter of researchers’ (and their funders’) agendas and interests will always shape the interpretation of women’s experiences. The findings bring clarity and evidence on determinant aspects that influence women’s wellbeing during and after a pregnancy. The underlying societal, cultural and economic factors on one side and the implications of morbidities on another are aspects that can hardly be quantified, but must be taken into consideration for programmatic initiatives. Our analysis is strong advocacy to not only amplify efforts to improve women’s positions in society, but also to consider investigation and measurement of maternal morbidity beyond physical and clinical indicators and explore the themes that have been highlighted here. We recommend that more high quality, analytical, theory-based, qualitative research be conducted that aims to allow the study of the complex experiences of maternal morbidity.

## Supporting information

S1 Appendix Search strategy for medline and Embase. (DOCX)

S2 Appendix Qualitative articles on women’s experiences of maternal morbidities descriptive characteristics. (DOCX)

S3 Appendix Prisma checklist. (DOC)
