# Supplementary material for: What maternal morbidities are and what they mean for women: A thematic analysis of twenty years of qualitative research in low and lower-middle income countries
Source: PLoS One. 2019 Apr 11;14(4):e0214199. doi: 10.1371/journal.pone.0214199 (PMC6459473; doi:10.1371/journal.pone.0214199)
Supplement: S1 Appendix — (DOCX) [file pone.0214199.s001.docx]

**S1 Appendix 1: Search strategy for Medline and Embase**

1. maternal or gestation$ or obstetric OR labo$r or pregnan$ or partum or antepartum or intrapartum or postpartum or post partum or antenatal or postnatal or post partal or puerperal or puerperium).mp.
2. ((maternal or gestation$ or obstetric labo$r or pregnan$ or partum$ or antepartum or intrapartum or postpartum$ or post partum or antenatal or postnatal or post partal or puerperal or puerperium) adj2 (health OR well$being OR morbid* OR ill* OR disorder$ OR disease$ OR disabilit* OR impairment OR survival)).ab,ti.
3. exp obstetric labor complications/
4. exp pregnancy complications/
5. ((pregnan$ or obstetric labo$r or maternal) and complication$).mp.
6. episiotomy/ or extraction, obstetrical/ or labor, induced/ or vaginal birth after cesarean/ or version, fetal/
7. or/ 3-6
8. ((ectopic or heterotopic or molar) and pregnancy).mp
9. (spontaneous abortion).mp.
10. or/8-9
11. 1 and (hyperten$ or eclampsia or pre-eclampsia or HELLP).mp
12. (uter$ and (hemorrhage or haemorrhage or prolapse or inversion or rupture or trauma or damage or laceration or tear or dehiscence)).mp
13. (placenta previa or placenta praevia).mp
14. exp Hemorrhage/
15. (haemorrhage or hemorrhage).mp
16. 1 and (or/12-15)
17. (puerperal infection$).mp
18. 1 and sepsis.mp
19. exp Mastitis/
20. (amnionitis or chorioamnionitis or membranitis or placentitis or endometritis or peritonitis or cervictis or vaginitis or trichomoniasis or Septic pelvic thrombosis or breast engorgement or ((breast or mammary or subareolar) and abscess)).mp
21. ((breast or uter$ or genit$ or perineal or pelvic) and infection$).mp
22. 1 and or/17-21
23. ((Hyperemesis or hyper-emesis) and gravidarum).mp
24. 1 and exp "Wounds and Injuries"/
25. 1 and (trauma or damage or laceration or tear or dehiscence or rupture).mp
26. or/23-25
27. exp Rectovaginal Fistula/ or exp urinary fistula/ or exp vesicovaginal fistula/ or exp vaginal fistula
28. exp pelvic organ prolapse/
29. ((obstetric or vesico-vaginal or vesicovaginal or vaginal or rectovaginal or urinary) and fistula).mp.
30. exp Urinary Incontinence
31. 1 and incontinence.mp
32. 1 and or/27-31
33. exp depression/ or exp Depressive Disorder/ or exp Stress Disorders, Post-Traumatic/ or exp Mental disorders/ or exp Anxiety/ or exp Anxiety Disorders/ or exp Psychotic Disorders/ or exp mental health/ or exp panic/
34. (((Mental or psycho$) and (ill$ or disorder or health)) or psychosis or anxiety or phobi$ or panic).mp
35. exp Suicide/
36. 1 and (or/33-35)
37. 1 and (exp bacterial infections/ or exp infection/ or exp virus diseases/ or exp parasitic diseases/)
38. 1 and (exp cardiovascular diseases/ or exp Respiratory Tract Diseases)
39. 1 and exp skin diseases/
40. 1 and exp Endocrine System Diseases/
41. 1 and exp Digestive System Diseases/
42. 1 and exp Female Urogenital Diseases/
43. 1 and (exp Hematologic Diseases/ or exp Lymphatic Diseases)
44. 1 and (exp Anemia/ or anemia.mp.)
45. 1 and exp Nervous System Diseases/
46. 1 and exp neoplasms/
47. 1 and exp Musculoskeletal Diseases/
48. 1 and (exp Metabolic Diseases/ or exp Nutrition Disorders/)
49. or/36-48
50. 2 or 7 or 10 or 11 or 16 or 22 or 26 or 32 or 49
51. Argentina or Bolivia or Brazil or Chile or Colombia or Ecuador or French Guiana or Guyana or Paraguay or Peru or Suriname or Uruguay or Venezuela or Mexico or Belize or Costa Rica or El Salvador or Guatemala or Honduras or Nicaragua or Panama or West Indies or Antigua or Bahamas or Barbados or Cuba or Dominica or Dominican Republic or Grenada or Guadeloupe or Haiti or Jamaica or Martinique or Antilles or (Saint Kitts and Nevis) or Saint Lucia or (Saint Vincent and the Grenadines) or Trinidad or Tobago or (Virgin Islands) or Kazakhstan or Kyrgyzstan or Tajikistan or Turkmenistan or Uzbekistan or Borneo or Brunei or Cambodia or East Timor or Indonesia or Laos or Malaysia or (Mekong Valley) or Myanmar or Burma or Philippines or Singapore or Thailand or Vietnam or Bangladesh or Bhutan or India or Nepal or Pakistan or Sri Lanka or China or Korea or Macao or Mongolia or Taiwan or Afghanistan or Bahrain or Iran or Iraq or Israel or Jordan or Kuwait or Lebanon or Oman or Qatar or Saudi Arabia or Syria or Turkey or United Arab Emirates or Yemen or Fiji or New Caledonia or Papua New Guinea or Vanuatu or Micronesia or Melanesia or Guam or Palau or Polynesia or Samoa or Tonga or Armenia or Azerbaijan or exp "Georgia (Republic)"/ or Albania or Estonia or Latvia or Lithuania or Bosnia or Herzegovina or Bulgaria or Belarus or Croatia or Czech Republic or Hungary or Macedonia or Moldova or Montenegro or Poland or Romania or Russia or Bashkiria or Dagestan or Slovakia or Slovenia or Ukraine or Cameroon or Central African Republic or Chad or Congo or (Democratic Republic of the Congo) or Equatorial Guinea or Gabon or Burundi or Djibouti or Eritrea or Ethiopia or Kenya or Rwanda or Somalia or Sudan or Tanzania or Uganda or Angola or Botswana or Lesotho or Malawi or Mozambique or Namibia or South Africa or Swaziland or Zambia or Zimbabwe or Benin or Burkina Faso or Cabo Verde Cote d'Ivoire or Gambia or Ghana or Guinea or Guinea-Bissau or Liberia or Mali or Mauritania or Niger or Nigeria or Senegal or Sierra Leone or Togo or Algeria or Egypt or Libya or Morocco or Tunisia or Comoros or Madagascar or Mauritius or Reunion or Seychelles or (third-world country) or (third world country) or (less developed) or caribbean.mp. or exp Caribbean Region/ OR pacific islands.mp. or exp Pacific Islands/ OR latin america.mp. or exp Latin America/ OR indian ocean islands.mp. or exp Indian Ocean Islands/ OR central america.mp. or exp Central America/ OR exp Asia/ or asia.mp. OR exp Africa/ or Africa.mp OR exp South America/ or south America.mp OR exp Far East/ or far east.mp or developing countries.mp. or exp Developing Countries/ or low income country.mp. or middle income country.mp. or subsaharan.mp.
52. ((("semi-structured" or semistructured or unstructured or informal or "in-depth" or indepth or "face-to-face" or structured or guide) adj3 (interview* or discussion* or questionnaire*))).ti,ab. or (focus group* or qualitative or ethnograph* or fieldwork or "field work" or "key informant").ti,ab. or interviews as topic/ or focus groups/ or narration/ or qualitative research/ or anthropology/ or anthropological/
53. 50 and 51 and 52
54. limit 53 to yr="1990-2015"
55. Limit 54 to humans
56. Limit 55 to female
