# Supplementary material for: What maternal morbidities are and what they mean for women: A thematic analysis of twenty years of qualitative research in low and lower-middle income countries
Source: PLoS One. 2019 Apr 11;14(4):e0214199. doi: 10.1371/journal.pone.0214199 (PMC6459473; doi:10.1371/journal.pone.0214199)
Supplement: S2 Appendix — (DOCX) [file pone.0214199.s002.docx]

S2 Appendix 2 Qualitative articles on women’s experiences of maternal morbidities descriptive characteristics

| # | Title | Journal/source | Author(s) last name, first initial | Publication date (year) | Location / Region | Specific morbidities | Qualitative data collection method | Analysis approach |
| --- | --- | --- | --- | --- | --- | --- | --- | --- |
| 1 | Postpartum health in a Dhaka slum | Social Science & Medicine | Uzma, A; Underwood, P;  Atkinson, D; Thackrah, R; | 1999 | Bangladesh, South Asia | post partum morbidity/health (general) | semi-structured questionnaire; focus group discussions | -- |
| 2 | Women’s Experiences of Utero-Vaginal Prolapse: A Qualitative Study from Tamil Nadu, India | Safe motherhood initiatives: critical issues | Ravindran, TKS; Savitri R;  Bhavani A | 2000 | India, South Asia | Uterine prolapse (utero-vaginal prolapse) | interviews | -- |
| 3 | Women's perceptions of postpartum depressive symptoms from an international perspective | International Nursing Perspectives | Horowitz, JA; Chang, SS; Das, S; Hayes, B | 2001 | India, South Asia | depression (post partum depression) | focus group discussions | Content analysis |
| 4 | Crossing the river: Khmer women’s perceptions of pregnancy and postpartum | Journal of Midwifery & Women’s Health | White, PM | 2002 | Cambodia, East Asia | general maternal morbidities (pregnancy and post-partum) | in-depth, semi structured interviews, focus group discussions | Theme based |
| 5 | Listening to mothers: Qualitative studies on motherhood and depression from Goa, India | Social Science & Medicine | Rodrigues, M; Patel, V; Jaswal, S; De Souza, N | 2003 | India, South Asia | depression, postnatal depression | in-depth interviews | Pre-established themes |
| 6 | Postnatal depression across countries and cultures: A qualitative study | British Journal of Psychiatry | Oates, MR; Cox, JL; Neema, S; Asten, P; Glangeaud-Freudenthal, N; Figueiredo, B; Gorman, LL; Hacking, S; Hirst, E; Kammerer, MH; Klier, CM; Seneviratne, G;  Smith, M; Sutter-Dallay, AL; Valoriani, V; Wickberg, B; Yoshida, K. | 2004 | Uganda, East Africa | depression, postnatal depression | interviews, focus group discussions | Textual analysis, thematic |
| 7 | Kaqchikel midwives, home births, and emergency obstetric referrals in Guatemala: contextualizing the choice to stay at home | Social Science & Medicine | Berry, NS | 2006 | Guatemala, Central America | Labour complications | ethnographic – participant observation, semi-structured interviews | Ethnographic |
| 8 | Chikotsa—Secrets, Silence, and Hiding:  Social Risk and Reproductive Vulnerability in Central Mozambique | Medical Anthropology Quarterly | Chapman, RR | 2006 | Mozambique, East Africa | general maternal and reproductive morbidity | ethnographic – in-depth interviews, participant observation, focus group discussions | Ethnographic |
| 9 | Treatment-seeking practices for malaria in pregnancy among rural women in Mukono District, Uganda | Journal of Biosocial Science | Mbonye, AK; Neema, S;  Magnussen, P | 2006 | Uganda, East Africa | Malaria | interviews, focus group discussions | Thematic based on the Health Belief Model |
| 10 | Children, pregnant women and the culture of malaria in two rural communities of Ghana | Anthropology and Medicine | Ahorlu, CK; Koram, KA;  Weiss, M G | 2007 | Ghana, West Africa | malaria in pregnancy | in-depth interviews, focus group discussions, free listing and rating, participatory mapping | Content analysis |
| 11 | Complex emotions, complex problems:  Understanding the experiences of perinatal depression among new mothers in urban Indonesia | Culture, Medicine and Psychiatry | Andajani-Sutjahjo, A; Manderson, L; Astbury, J | 2007 | Indonesia, East Asia and Pacific | Postpartum depression | in-depth interviews | Thematic analysis |
| 12 | A qualitative investigation into knowledge, beliefs, and practices surrounding mastitis in sub-Saharan Africa: what implications for vertical transmission of HIV? | BMC Public Health | De Allegri, M; Sarker, M;  Hofmann, J; Sanon, M;  Bohler, T | 2007 | Burkina Faso, West Africa | mastitis | in-depth interviews, focus group discussions | Inductive, analyst triangulation |
| 13 | Women’s Descriptions of Postpartum Health Problems: Preliminary Findings from Matlab, Bangladesh | Journal of Midwifery & Women’s Health | Sibley LM; Blum LS; Kalim N; Hruschka D; Edmonds JK; Koblinsky M | 2007 | Bangladesh, South Asia | post-partum health problems | semi-structured interviews | Descriptive |
| 14 | Experiences of women seeking medical care for obstetric fistula in Eritrea: Implications for prevention, treatment, and social reintegration | Global Public Health | Turan, JM; Johnson, K;  Polan, ML | 2007 | Eritrea, East Africa | obstetric fistula | open ended interviews, semi-structured interviews | Simple descriptive analyses |
| 15 | Post-partum depression in Kinshasa, Democratic Republic of Congo: Validation of a concept using a mixed-methods cross-cultural approach | Tropical Medicine and International Health | Bass, JK; Ryder, RW; Lammers, MC; Mukaba, TN; Bolton, PA | 2008 | Democratic Republic of Congo, Central Africa | depression, post-partum depression | interviews, free listing | Consolidation, ranking |
| 16 | Perception of bleeding as a danger sign during pregnancy, delivery, and the postpartum period in rural Nepal | Qualitative Health Research | Matsuyama, A;  Moji, K | 2008 | Nepal, South Asia | bleeding | interviews, case histories, free-listing, ranking, pile-sorting | Grounded theory, inductive approach, content analysis |
| 17 | Health and Social Problems Encountered by Treated and Untreated Obstetric Fistula Patients in Rural Ethiopia | Journal of Obstetrics and Gynaecology Canada, Women's Health | Muleta, M, Hamlin, EC; Fantahun, M; Kennedy, RC; Tafesse, B | 2008 | Ethiopia, East Africa | obstetric fistula | in-depth interviews | Thematic analysis |
| 18 | Postnatal mental distress in relation to the sociocultural practices of childbirth: An exploratory qualitative study from Ethiopia | Social Science & Medicine | Hanlon, C; Whitley, R;  Wondimagegn, D; Alem, A; Prince, M | 2009 | Ethiopia, East Africa | depression (postnatal depression) | in-depth interviews, focus group discussions | Inductive analysis, theme based |
| 19 | Postpartum Haemorrhage and Eclampsia: Differences  in Knowledge and Care-seeking Behaviour in  Two Districts of Bangladesh | Journal of Health, Population and Nutrition | Kalim, N; Anwar, I; Khan, J; Blum, L; Botlero, R; Koblinsky, M | 2009 | Bangladesh, South Asia | Postpartum haemorrhage and eclampsia (near miss) | in-depth interviews, free-listing, rating exercises, hypothetical case scenarios | Content analysis |
| 20 | Cultural theories of postpartum bleeding in Matlab, Bangladesh: implications for community health intervention | Journal of Health, Population & Nutrition | Sibley, LM; Hruschka, D;  Kalim, N; Khan, J; Paul, M;  Edmonds, JK; Koblinsky, MA | 2009 | Bangladesh, South Asia | post partum bleeding | semi-structured interviews | -- |
| 21 | The lived experience of Malawian women with obstetric fistula | Culture, Health & Sexuality: An International Journal  for Research, Intervention and Care | Yeakey, M; Chipeta, E; Taulo, F; Tsui, A | 2009 | Malawi, East Africa | Obstetric fistula | open ended interviews | Phenomenologi-cal approach |
| 22 | Understanding women's experiences of distress during pregnancy in Dar es Salaam, Tanzania | Tanzania Journal of Health Research | Kaaya, SF; Mbwambo, JK;  Smith Fawzi, MC; Van Den Borne, H; Schaalma, H; Leshabari, MT | 2010 | Tanzania, East Africa | mental distress | unstructured interviews | Narrative analysis |
| 23 | Antenatal anxiety in pregnant women from rural Cambodia | British Journal of Midwifery | MacLellan, J | 2010 | Cambodia, East Asia | mental distress, antenatal anxiety | in-depth interviews | Grounded theory |
| 24 | The social contexts of depression during motherhood: a study of Explanatory Models in Vietnam | Journal of Affective Disorders | Niemi, ME; Falkenberg, T;  Nguyen, MT; Patel, V;  Faxelid, E; | 2010 | Vietnam, East Asia | depression and post natal depression | semi structured interviews, explanatory models | Conventional content analysis |
| 25 | Marginal matters: Pregnancy loss as a social event | Social Science and Medicine | van der Sijpt, E | 2010 | Cameroon, Central Africa | pregnancy loss | participant observation, informal conversations, interviews | Ethnographic |
| 26 | Perils to Pregnancies: On Social Sorrows and Strategies Surrounding Pregnancy Loss in Cameroon | Medical Anthropology Quarterly | van der Sijpt, E; Notermans, C | 2010 | Cameroon. Central Africa | reproductive loss | Ethnographic – participant observation, in depth interviews, life & reproductive histories, focus group discussions, body mapping | Ethnographic, systematic content analysis |
| 27 | Beyond body counts: A qualitative study of lives and loss in Burkina Faso after ‘near-miss’ obstetric complications | Social Science & Medicine | Storeng, KT; Murray, SF;  Akoum, MS; Ouattara, F; Filippi, V. | 2010 | Burkina Faso, West Africa | near miss | ethnographic, in-depth interviews | Interpretative and thematic |
| 28 | The psychosocial impact of vesico-vaginal fistula in Niger | Arch Gynecol Obstet | Alio, AP; Merrel, L; Roxburgh, K; Clayton, KB; Marty, PJ; Bomboka, L; Traore, S; Salihu, HM | 2011 | Niger, West Africa | obstetric fistula | in-depth interviews | Ethnographic inductive approach and the ecological  model, theme based |
| 29 | Delays in recognition of and care seeking response to prolonged labor in Bangladesh | SSM | Head, S; Yount, K; Sibley, L | 2011 | Bangladesh, South Asia | prolonged labour | interviews, group interviews (In particular: Integrated Illness History interviews) | -- |
| 30 | The pathway of obstructed labour as perceived by communities in south-western Uganda: a grounded theory study | Global Health Action | Kabakyenga, JK; Ostergren, PO; Emmelin, M; Kyomuhendo, P; Odberg Pettersson, K; | 2011 | Uganda, East Africa | obstructed labour | focus group discussions | Grounded theory |
| 31 | Cultural Perceptions of Maternal Illness  among Khmer Women in Krong Kep,  Cambodia | vis-à-vis: Explorations in Anthropology | Montesanti, S. | 2011 | Cambodia, East Asia | maternal illness, general | semi-structured interviews | -- |
| 32 | “I am nothing”: experiences of loss among women  suffering from severe birth injuries in Tanzania | BMC Women’s Health | Mselle, L; Moland, K; Evjen-Olsen, B; Mvungi, A; Kohi, T | 2011 | Tanzania, East Africa | obstetric fistula | semi-structured interviews, focus group discussions | Content data analysis framework |
| 33 | Experiences with fistula repair surgery among women and families in Malawi | Global Public Health | Yeakey, MP; Chipeta, E;  Rijken, Y; Taulo, F; Tsui, AO | 2011 | Malawi, East Africa | obstetric fistula | Open ended interviews, including follow up interviews | Inductively derived themes and domains |
| 34 | Still living with fistula: an exploratory study of the experience  of women with obstetric fistula following corrective surgery  in West Pokot, Kenya | Reproductive Health Matters | Khisa, A; Nyamongo, I | 2012 | Kenya, East Africa | fistula and surgical repair | in-depth interviews, focus group discussions | Grounded theory, thematic analysis |
| 35 | Capitals diminished, denied, mustered and deployed: A qualitative longitudinal study of women's four year trajectories after acute health crisis, Burkina Faso | Social Science & Medicine | Murray, SF; Akoum, MS;  Storeng, KT | 2012 | Burkina Faso, West Africa | near miss (acute health crisis in pregnancy /delivery) | in-depth interviews, qualitative longitudinal research (QLR) | Thematic analysis |
| 36 | Violence against women with chronic maternal disabilities in rural Bangladesh | Journal of Health, Population & Nutrition | Naved, RT; Blum, LS;  Chowdhury, S; Khan, R;  Bilkis, S; Koblinsky, M | 2012 | Bangladesh, South Asia | uterine prolapse, stress incontinence, obstetric fistula | in-depth interviews | Content analysis |
| 37 | Absence and reliance: Liberian women's experience of vaginal fistula | African Journal of Midwifery & Women's Health | Söderbäck, M; Wilhelmsson, E; Häggström-Nordin, E | 2012 | Liberia, West Africa | obstetric fistula | ethnographic – naturalistic observation, group & individual conversations | Reflexive analysis, theme based |
| 38 | The influence of socio-cultural interpretations of pregnancy threats on health-seeking behavior among pregnant women in urban Accra, Ghana | BMC Pregnancy & Childbirth | Dako-Gyeke, P; Aikins, M;  Aryeetey, R; McCough, L;  Adongo, PB | 2013 | Ghana, West Africa | threats to pregnancy which produced anxiety, mental distress | in-depth interviews, focus group discussions | Thematic analysis |
| 39 | Local illness concepts and their relevance for the prevention and control of malaria during pregnancy in Ghana, Kenya and Malawi: findings from a comparative qualitative study | Malaria Journal | Menaca, A; Pell, C;  Manda-Taylor, L; Chatio, S; Afrah, N; Were, F; Hodgson, A; Ouma, P; Kalilani, L; Tagbor, H;  Pool, R | 2013 | Ghana, West Africa; Kenya and Malawi, East Africa | malaria | anthropological approach – free-listing  and sorting, in-depth interviews, focus group discussions,  participant observation | Pre-established and emergent themes, comparative |
| 40 | Lived Experiences of Ghanaian Women  With Obstetric Fistula | Health Care for Women International | Mwini-Nyaledzigbor, P; Agana, A; Pilkington, F | 2013 | Ghana, West Africa | Obstetric fistula | interviews | Content analysis |
| 41 | Survivors' understanding of vulnerability and resilience to maternal near-miss obstetric events in Uganda | International Journal of Gynecology and Obstetrics | Kaye, DK; Kakaire, O;  Nakimuli, A; Mbalinda, SN; Osinde, MO; Kakande, N | 2014 | Uganda, East Africa | near miss | in-depth interviews | Phenomenological, thematic |
| 42 | ‘This is normal during pregnancy’: A qualitative study of anaemia-related perceptions and practices among pregnant women in Mumbai, India | Midwifery | Chatterjee, N., Fernandes, G. | 2014 | India, South Asia | anaemia | in-depth interviews, focus group discussions | Thematic analysis |
| 43 | Understanding psychological distress among mothers in rural Nepal: A qualitative grounded theory exploration | BMC Psychiatry | Clarke, K; Saville, N; Bhandari, B; Giri, K; Ghising, M; Jha, M; Jha, S; Magar, J; Roy, R; Shrestha, B; Thakur, B; Tiwari, R; Costello, A; Manandhar, D; King, M; Osrin, D; Prost, A | 2014 | Nepal, South Asia | mental distress, psychological distress | semi structured interviews, focus group discussions | Grounded theory |
| 44 | Lived experiences of women who developed  uterine rupture following severe obstructed labor  in Mulago hospital, Uganda | Reproductive Health | Kaye DK; Kakaire O; Nakimuli A; Osinde MO; Mbalinda SN; Kakande N | 2014 | Uganda, East Africa | traumatic maternal events (uterine rupture following obstructed labor) and their outcomes (near miss) | in-depth interviews | Thematic analysis |
| 45 | The unfortunate sufferer: discursive dynamics around pregnancy loss in Cameroon | Medical anthropology | van der Sijpt, E. | 2014 | Cameroon, West / Central Africa | pregnancy loss | Ethnographic – participant observation, in-depth interviews | ethnographic |
| 46 | “I can't stop worrying about everything” – Experiences of rural Bangladeshi women during the first postpartum months | International Journal of Qualitative Studies on Health & Well-Being | Edhborg, M;  Nasreen, HE;  Kabir, ZN | 2015 | Bangladesh, South Asia | mental distress (worrying) | open narrative interviews | Inductive content analysis |
| 47 | Perinatal distress and depression in Malawi: an exploratory qualitative study of stressors, supports and symptoms | Archives of Women's Mental Health | Stewart, R; Umar, E;  Gleadow-Ware, S; Creed, F; Bristow, K | 2015 | Malawi, East Africa | depression, anxiety, distress in pregnancy and post partum (perinatal period) | focus group discussions | Thematic analysis |
| 48 | Consequences of severe obstetric complications on women's health in Morocco: Please, listen to me! | Tropical Medicine and International Health. | Assarag, B;  Dujardin, B;  Essolbi, A;  Cherkaoui, I;  De Brouwere, V | 2015 | Morocco, North Africa | Near miss | in-depth interviews | - |
| 49 | Sick Healers: Chronic Affliction and the Authority of Experience at an Ethiopian Hospital | American Anthropologist | Hannig, A | 2015 | Ethiopia, East Africa | Fistula and surgical repair | ethnographic | Ethnographic |
| 50 | Quality of life of Ethiopian women after fistula repair: implications on rehabilitation and social reintegration policy and programming | Culture, health & sexuality | Donnelly, K; Oliveras, E;  Tilahun, Y; Belachew, M;  Asnake, M | 2015 | Ethiopia, East Africa | obstetric fistula | in-depth interviews | Grounded theory, narrative thematic techniques |
| 51 | Giving Voice to the Experiences of Rwandan Women with Urogenital Fistula | Annals of Global Health | Kay, A; Nishimwe, A;  Hampton, BS | 2015 | Rwanda, East Central Africa | obstetric fistula | interview | Analysis of salient themes |
| 52 | Obstetric Fistula "Disease" and Ensuing Care: Patients' Views in West-Africa | African journal of reproductive health | Maulet, N; Berthe, A;  Traore, S; Macq, J; | 2015 | Mali and Niger, West Africa | obstetric fistula | semi-structured interviews, observation, rapport establishment | Use of an emerging analytical framework |
| 53 | Living with constant leaking of urine and odour: Thematic analysis of socio-cultural experiences of women affected by obstetric fistula in rural Tanzania | BMC Women's Health | Mselle, LT;  Kohi, TW; | 2015 | Tanzania, East Africa | obstetric fistula | semi-structured interviews, focus group discussions | Thematic analysis |
| 54 | What is in a name? Causative explanatory models of postpartum psychosis among patients and caregivers in India | International Journal of Social Psychiatry | Thippeswamy, H; Dahale, A; Desai, G; Chandra, PS | 2015 | India, South Asia | post-partum psychosis | open ended interviews open using the Short Explanatory Model Interview (SEMI)] | Content analysis |
| 55 | Experiences of social support among women presenting for obstetric fistula repair surgery in Tanzania | International Journal of Women's Health | Dennis, AC; Wilson, SM;  Mosha, MV; Masenga, GG; Sikkema, KJ; Terroso, KE; Watt, MH | 2016 | Tanzania, East Africa | obstetric fistula | in-depth interviews | Narrative and analytical memos, thematic |
| 56 | Long-term outcomes for women after obstetric fistula repair in Lilongwe, Malawi: A qualitative study | BMC Pregnancy and Childbirth | Drew, LB; Wilkinson, JP;  Nundwe, W; Moyo, M;  Mataya, R; Mwale, M;  Tang, JH | 2016 | Malawi, East Africa | obstetric fistula | in-depth interviews | Content analysis |
| 57 | Huwa Rog, Parhej, and Desi Dawai: Women's Perceptions of Postpartum Maternal Morbidity and Care in Rajasthan, India | Qualitative health research | Iyengar, K; Pelto, P;  Iyengar, SD | 2016 | India, South Asia | general post-partum illness (severe and less severe) | semi-structured interviews, case interviews, free listing | Coding on key topics, triangulations of concepts |
| 58 | Sources of support for women experiencing obstetric fistula in northern Ghana: A focused ethnography | Midwifery | Sullivan, G; O'Brien, B;  Mwini-Nyaledzigbor, P | 2016 | Ghana, West Africa | obstetric fistula | in-depth interviews, non-participant observation | Non linear reflexive process to develop conceptual models |
| 59 | What makes pregnant workers sick: Why, when, where and how? An exploratory study in the ready-made garment industry in Bangladesh | Reproductive Health | Akhter, S; Rutherford, S; Chu, C | 2017 | Bangladesh, South Asia | general pregnancy ill health and morbidity | in-depth interviews | Framework analysis |
| 60 | Understanding coping strategies during pregnancy and the postpartum period: A qualitative study of women living with HIV in rural Uganda | BMC Pregnancy and Childbirth | Ashaba, S; Kaida, A; Burns, BF; O'Neil, K;  Dunkley, E; Psaros, C; Kastner, J; Tsai, AC; Bangsberg, DR; Matthews, LT | 2017a | Uganda, East Africa | HIV | semi-structured interviews | Content analysis, thematic analysis |
| 61 | Psychosocial challenges facing women living with HIV during the perinatal period in rural Uganda | PLoS ONE | Ashaba, S; Kaida, A; Coleman, JN; Burns, BF; Dunkley, E; O'Neil, K; Kastner, J; Sanyu, N; Akatukwasa, C; Bangsberg, DR; Matthews, LT; Psaros, C | 2017b | Uganda, East Africa | HIV | semi-structured interviews, in-depth interviews | Content analysis |
| 62 | I am a person but I am not a person': Experiences of women living with obstetric fistula in the central region of Malawi | BMC Pregnancy and Childbirth | Changole, J;  Thorsen, VC;  Kafulafula, U | 2017 | Malawi, East Africa | obstetric fistula | semi-structured interviews | Thematic analysis, theory of stigmatization |
| 63 | Impact of surgery on quality of life of women with obstetrical fistula: a qualitative study in Burkina Faso | International Urogynecology Journal | Desalliers, J;  Pare, ME;  Kouraogo, S;  Corcos, J | 2017 | Burkina Faso, West Africa | obstetric fistula | semi-directed interviews, principles of participative action research (PAR) | Grounded theory |
| 64 | Development and preliminary validation of a post-fistula repair reintegration instrument among Ugandan women | Reproductive Health | El Ayadi, A; Nalubwama, H; Barageine, J; Neilands, TB; Obore, S; Byamugisha, J; Kakaire, O;  Mwanje, H; Korn, A;  Lester, F; Miller, S | 2017 | Uganda, East Africa | obstetric fistula | in-depth interviews, focus group discussions | Thematic analysis |
| 65 | Unsettling the fistula narrative: cultural pathology, biomedical redemption, and inequities of health access in Niger and Ethiopia | anthropology and Medicine | Heller, A;  Hannig, A | 2017 | Niger, West Africa and Ethiopia, East Africa | obstetric fistula | ethnographic | Ethnographic |
| 66 | Experiences of mothers with gestational diabetes in Ghana | Journal of Research in Nursing and Midwifery | Mensah, GP; Affram, CK; Richter, S; Banful, R | 2017 | Ghana, West Africa | GDM | semi-structured interviews | The Colaizzi method – descriptive phenomeno-logical approach |
| 67 | I stayed with my illness': A grounded theory study of health seeking behaviour and treatment pathways of patients with obstetric fistula in Kenya | BMC Women's Health | Khisa, AM; Omoni, GM;  Nyamongo, IK; Spitzer, RF | 2017a | Kenya, East Africa | obstetric fistula | interviews | Grounded theory |
| 68 | Understanding the lived experience of women before and after fistula repair: a qualitative study in Kenya | BJOG: An International Journal of Obstetrics and Gynaecology | Khisa, W; Wakasiaka, S;  McGowan, L; Campbell, M; Lavender, T | 2017b | Kenya, East Africa | obstetric fistula | semi-structured interviews | Hermeneutic interpretative phenomen-ology |
| 69 | Women's experiences of having had, and recovered from, eclampsia at a tertiary hospital in Tanzania | Women and Birth | Mukwenda, AM;  Mbekenga, CK; Pembe, AB; Olsson, P | 2017 | Tanzania, East Africa | eclampsia | semi-structured interviews | Thematic analysis |
| 70 | Injured bodies, damaged lives: Experiences and narratives of Kenyan women with obstetric fistula and Female Genital Mutilation/Cutting | Reproductive Health | Mwanri, L; Gatwiri, GJ; | 2017 | Kenya, East Africa | obstetric fistula | semi-structured interviews | Social Network Framework and a feminist analysis |
| 71 | A qualitative study of pregnancy-related anxiety among women in Tanzania | BMJ Open | Rosario, MK;  Premji, SS;  Nyanza, EC;  Bouchal, SR;  Este, D | 2017 | Tanzania, East Africa | anxiety | semi-structured interviews | The Colaizzi method – descriptive phenomenologi-cal approach |
